# Sphingosine 1-Phosphate Receptor Blockade Affects Pro-Inflammatory Bone Marrow-Derived Macrophages and Relieves Mouse Fatty Liver Injury

**DOI:** 10.3390/ijms20194695

**Published:** 2019-09-22

**Authors:** Jingjing Yang, Na Chang, Le Yang, Xiaofang Ji, Xuan Zhou, Lei Tian, Yuehan Ma, Yuanru Yang, Yuran Liu, Lin Yang, Liying Li

**Affiliations:** Department of Cell Biology, Municipal Laboratory for Liver Protection and Regulation of Regeneration, Capital Medical University, Beijing 100069, Chinachangna@ccmu.edu.cn (N.C.); , , , , , , , yang_lin@ccmu.edu.cn (L.Y.)

**Keywords:** sphingosine 1-phosphate, inflammation, macrophage, methionine-choline-deficient and high-fat diet

## Abstract

Fatty liver injury is characterized by liver fat accumulation and results in serious health problems worldwide. There is no effective treatment that reverses fatty liver injury besides etiological therapy. Inflammation is an important macrophage-involving pathological process of liver injury. Here, we investigated the role of sphingosine 1-phosphate receptors (S1PRs) in fatty liver injury and explored whether S1PR_2/3_ blockade could cure fatty liver injury. A methionine-choline-deficient and a high-fat (MCDHF) diet was used to induce fatty liver injury, and the number of macrophages was evaluated by flow cytometry. Gene expressions were detected using RT-qPCR and cytometric bead array. In MCDHF-diet-fed mice, pro-inflammatory factor expressions were upregulated by fatty liver injury. The S1P level and S1PR_2/3_ expressions were significantly elevated. Moreover, increased S1P level and S1PR_2/3_ mRNA expressions were positively correlated with pro-inflammatory factor expressions in the liver. Furthermore, the number of pro-inflammatory macrophages (iMφ) increased in injured liver, and they were mainly bone-marrow-derived macrophages. In vivo, S1PR_2/3_ blockade decreased the amount of iMφ and inflammation and attenuated liver injury and fibrosis, although liver fat accumulation was unchanged. These data strongly suggest that anti-inflammatory treatment by blocking the S1P/S1PR_2/3_ axis attenuates fatty liver injury, which might serve as a potential target for fatty liver injury.

## 1. Introduction

Fatty liver injury is the most common characteristic for alcoholic liver disease or non-alcoholic liver disease (NAFLD). It has been reported that steatohepatitis, which is present in fatty liver injury patients, can progress to fibrosis, cirrhosis, and hepatocellular carcinoma [1]. There is no effective treatment for reversing fatty liver injury besides etiological therapy. It has been reported that patients with fatty liver injury are characterized by hepatic inflammation [2,3], which is considered as the major driver of liver fibrogenesis [4,5]. In this process, macrophages play an important role. Liver macrophages consist of liver-resident Kupffer cells (KCs) and bone-marrow (BM)-derived monocyte/macrophages (BMMs) [6,7,8]. Our previous studies have shown that BMMs play a key role in liver injury [9]. Recruiting macrophages are activated as pro-inflammatory macrophages (iMφ) by various stimuli associated with metabolic disease [8]. Pro-inflammatory macrophages produce pro-inflammatory cytokines and chemokines, such as tumor necrosis factor (TNF)-α, macrophage inflammatory protein (MIP)-1β (also known as chemokine (C-C motif) ligand (CCL) 4), and monocyte chemotactic protein (MCP)-1 (also known as CCL2) [10]. Importantly, the high levels of MIP-1β, TNF-α, and MCP-1 in serum and liver tissue are positively correlated with hepatic injury and fibrogenesis [3,4,5]. Strategies for regulating macrophage function markedly attenuate hepatic injury and fibrosis in experimental models [11]. For these reasons, we ask whether there is a new target to regulate iMφ function and to be an effective therapy for fatty liver injury.

Sphingosine 1-phosphate (S1P), one of the most important lipid mediators, is formed by sphingomyelin metabolism [12]. In cells, the concentration of S1P is regulated by its synthases (sphingosine kinase (SphK), including SphK_1_ and SphK_2_) and degradation enzymes (S1P phosphatase (Sgpp1) and S1P lyase (Sgpl1)) [13]. During liver injury, erythrocytes, platelets, and hepatic myofibroblasts (the extracellular matrix (ECM)-producing cells in liver fibrogenesis) are main sources of S1P in liver [14,15]. Studies have shown that most S1P biological functions are mediated by its receptors. The S1P receptors (S1PRs) are classified into five types, named S1PR_1–5_. It has been reported that S1P and S1PRs are important regulators in various physiological or pathological processes [12,16,17]. In consideration of the importance of S1PRs in diseases, drugs targeting S1PRs have been developed, such as FTY720 (fingolimod, broad-spectrum S1PR modulator), BAF312 (siponimod, targeting S1PR_1/5_), PRC1063 (ozanimod, targeting S1PR_1/5_), and APD334 (etrasimod, targeting S1PR_1,4,5_) [16,18]. Among these drugs, FTY720 has been approved by the United States Food and Drug Administration and the others are also performing clinical tests [16,19,20,21,22]. But these drugs are mainly used to treat multiple sclerosis and ulcerative colitis. On the other hand, there are very few drugs specific targeting S1PR_2/3_, which are reported as key regulators in liver injury. The S1PR_2/3_ are widely expressed in various kinds of hepatic cells. In a recent study, researchers have reported that S1PR_2_ blockade relieves liver injury in bile duct ligation mice [23]. Our previous studies have also proved that S1PR_2/3_ are involved in BMMs migration through G(α)_i/o_/PI3K/Rac1 signaling pathway and S1PR_2/3_ antagonists decrease BMMs recruitment in cholestatic-injured liver. At the same time, S1PR_2/3_ regulate pro-inflammatory factor expressions in BMMs by activating G(α)_i/o_/PI3K/JNK signaling pathway in vitro [9,24]. For these reasons, we explored the role of S1PR_2/3_ in fatty liver injury and asked whether S1PR_2/3_ could be effective therapeutic targets for inhibiting inflammation and alleviating liver fibrosis in vivo.

Here, we investigated the effects of S1PR_2/3_ blockade on methionine-choline-deficient and high-fat (MCDHF) diet-induced fatty liver injury. The S1PR_2/3_ blockade decreased the number pro-inflammatory BMMs and pro-inflammatory factor release in vivo. Furthermore, S1PR_2/3_ blockade alleviated fibrosis in MCDHF diet-fed mice. Our present work identified S1PR_2/3_ as essential mediators in macrophage-involved liver inflammation and fibrosis, and as novel therapeutic targets of fatty liver injury.

## 2. Results

### 2.1. Mouse Fatty Liver Model Induced by MCDHF Diet Shows Progressive Hepatic Steatosis, Inflammation, and/then Fibrosis

**Figure 1:**
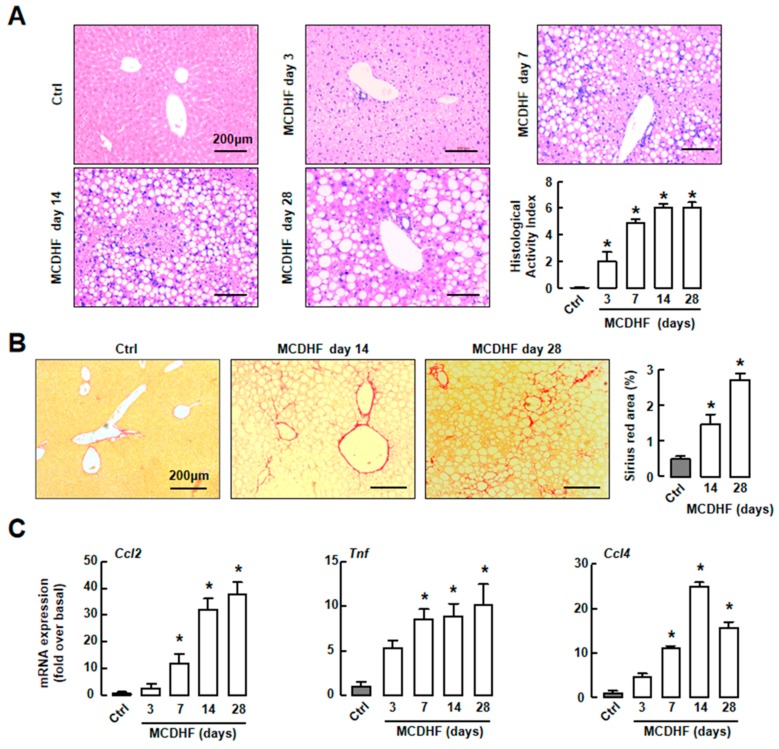
Characterization of hepatic steatosis, inflammation, and fibrosis during methionine-choline-deficient and high-fat diet (MCDHF) diet-induced mouse fatty liver damage. (**A**) The extent of inflammation and steatosis in the livers of MCDHF diet-fed mice were detected by H&E staining from day 3 to day 28. Scale bars = 200 μm. (**B**) The extent of hepatic fibrosis in the livers of MCDHF diet-treated mice was detected by Sirius Red staining. Scale bars = 200 μm. (**C**) Hepatic mRNA expressions of MCP-1 (*Ccl2*), TNF-α (*Tnf*), and MIP-1β (*Ccl4*) were determined by RT-qPCR. Data are presented as the means ± SEM. * *p* < 0.05 versus the control group (*n* = 6 for each group in each experiment).

To monitor the process of fatty liver injury, we set-up an MCDHF diet-induced mouse fatty liver model. We first employed H&E or Sirius Red staining to determine the morphologic changes of livers. We observed a significant increase of steatosis, hepatocellular ballooning, and inflammatory foci in liver at as early as day 7 (Figure 1A). Histology activity index was used to evaluate the degrees of liver injury. The scores were increased obviously after MCDHF diet feeding, indicating that abnormal fat accumulation of hepatocytes induced inflammation significantly (Figure 1A). The results of Sirius Red staining showed that collagen fiber deposition was observed in mouse liver after 14 days of MCDHF diet feeding (Figure 1B). These results suggested the successful establishment of MCDHF diet-induced mouse fatty liver injury model.

Then, we employed RT-qPCR to study the expressions of representative pro-inflammatory factors, including MCP-1 (*Ccl2*), TNF-α (*Tnf*), and MIP-1β (*Ccl4*). In injured livers, these pro-inflammatory factor levels were markedly upregulated at day 7 and continued to increase along with liver injury progression (Figure 1C). These data indicated that inflammation accompanied the whole fatty liver injury process, suggesting the importance of inflammation in fatty liver injury.

### 2.2. S1P/S1PR_2/3_ Axis was Associated with Pro-Inflammatory Factor Production in MCDHF Diet-Treated Livers

**Figure 2:**
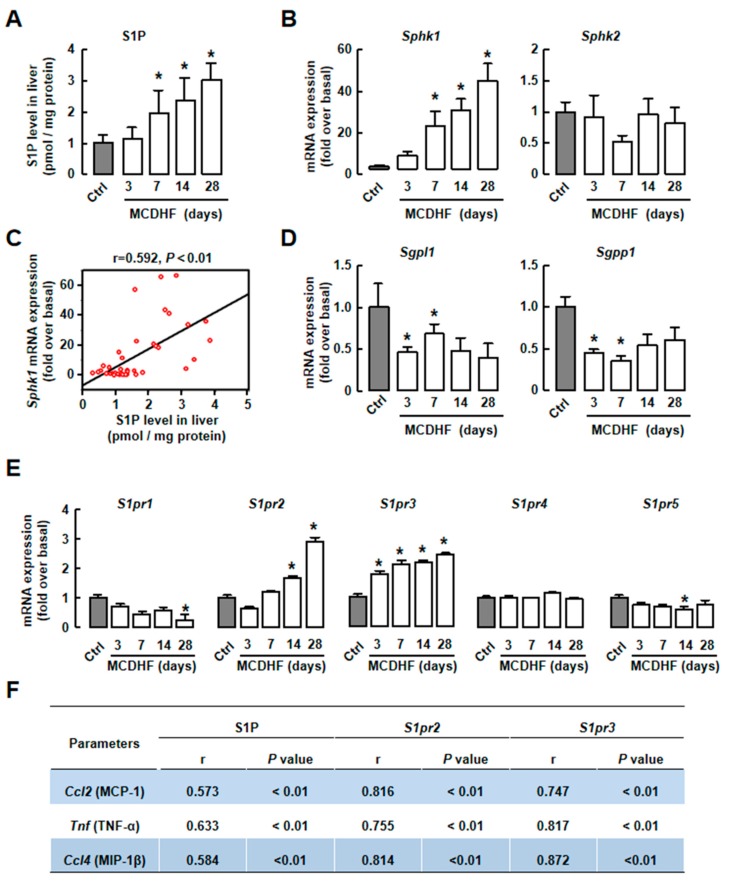
The MCDHF-diet-induced hepatic inflammation was associated with increasing levels of S1P/S1PR_2/3_. (**A**) The S1P level in liver tissues was analyzed by ELISA. (**B**) The mRNA expressions of *SphK1* and *SphK2* in MCDHF-diet-fed mice. (**C**) The relationship between S1P level and *SphK1* mRNA expression in liver were analyzed by regression analysis. (**D**) Hepatic mRNA expressions of S1P lyase and S1P phosphatase were determined by RT-qPCR. (**E**) Hepatic mRNA expressions of *S1pr1-5* were determined by RT-qPCR. (**F**) The relationship between hepatic S1P level or *S1pr2/3* mRNA expressions and pro-inflammatory factor expressions were analyzed by regression analysis (*n* = 42). Correlation coefficients (*r*). Data are presented as the mean ± SEM. *p < 0.05 versus control group (*n* = 6 for each group in each experiment).

Since previous studies have reported that the S1P/S1PR axis plays a critical role in liver injury [9,25], we checked S1P production in MCDHF-diet-induced fatty liver injury. The result of ELISA showed that S1P level was significantly increased at day 7, with a maximal increase (~2.94-fold) at day 28 (Figure 2A). Next, the enzymes related to S1P production were detected. The expression of *Sphk1* was upregulated, while *Sphk2* expression was unchanged (Figure 2B). The expressions of *Sgpl1* and *Sgpp1* were decreased (Figure 2D). Furthermore, there was positive correlation between S1P level and SphK1 mRNA expression in MCDHF diet-treated livers (Figure 2C), suggesting that upregulated SphK1 was the main reason for high S1P levels in liver.

S1PR expressions were also executed. The result of RT-qPCR showed that the expressions of *S1pr2* and *S1pr3* were markedly increased in injured livers, while *S1pr1, S1pr4,* and *S1pr5* were unchanged in this process (Figure 2E). Moreover, there were positive correlations of pro-inflammatory factor mRNA expressions with S1P levels or S1PR_2/3_ mRNA expressions in injured livers (Figure 2F). We also separated macrophages from hepatic non-parenchymal cells (NPCs) and compared S1PRs expression in macrophages and NPCs (besides macrophages). The results of RT-qPCR showed that S1PR_2_ levels were much higher in macrophages than other NPCs in injured livers (Supplementary Materials Figure S1A). These results suggest that the S1P/S1PR_2/3_ axis, which is obviously changed in fatty liver, might be associated with hepatic inflammation in MCDHF-diet-induced liver injury.

### 2.3. Blockade of S1PR_2/3_ Alleviates Hepatic Inflammation by Decreasing the Number of Pro-Inflammatory BMMs and Inflammatory Factor Expression in Fatty Liver Injury

**Figure 3:**
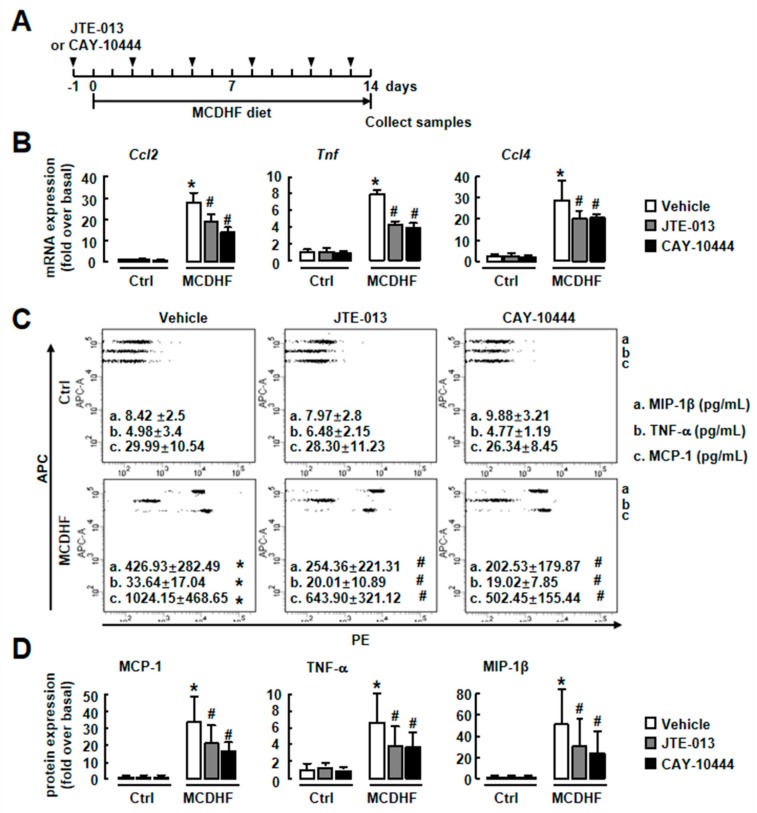
S1PR_2/3_ blockade decreased hepatic inflammation in the MCDHF-diet-fed mice. (**A**) Scheme of the mouse model. (**B**) The mRNA expressions of pro-inflammatory factors in the liver from vehicle and JTE-013- and CAY-10444-treated mice after 14 days of control or MCDHF diet feeding. (**C**) The hepatic protein expressions of pro-inflammatory factors were detected by CBA. The representative flow cytometric images are shown. The signals of the APC channel, which was formed by specific antibody-coated beads of different fluorescence intensity, showed different pro-inflammatory factors as marked. The signals of the PE channel showed the levels of pro-inflammatory factors in each sample. (**D**) Quantification of CBA. The concentrations of pro-inflammatory factors were calculated and compared with the control group. Data are presented as the mean ± SEM. * *p* < 0.05 versus the control group. # *p* < 0.05 versus MCDHF diet group (*n* = 6 for each group in each experiment).

To examine whether S1PR_2/3_ blockade inhibits inflammation in vivo, we fed mice for 14 days with an MCDHF diet in the absence or presence of JTE-013 or CAY-10444 (specific antagonist of S1PR_2_ or S1PR_3_, respectively). The mRNA and protein expressions of pro-inflammatory factors (MCP-1, TNF-α, and MIP-1β) were elevated in mice with MCDHF diet, and JTE-013 or CAY-10444 decreased their expressions (Figure 3). We also treated MCDHF-diet-fed mice with both JTE-013 and CAY-10444. The combined blockade of S1PR_2/3_ inhibited hepatic inflammation but did not show additive effects (Supplementary Materials Figure S1C–E). These results indicate S1PR_2/3_ blockade decreases pro-inflammatory factor production and alleviates hepatic inflammation efficiently in fatty liver injury.

**Figure 4:**
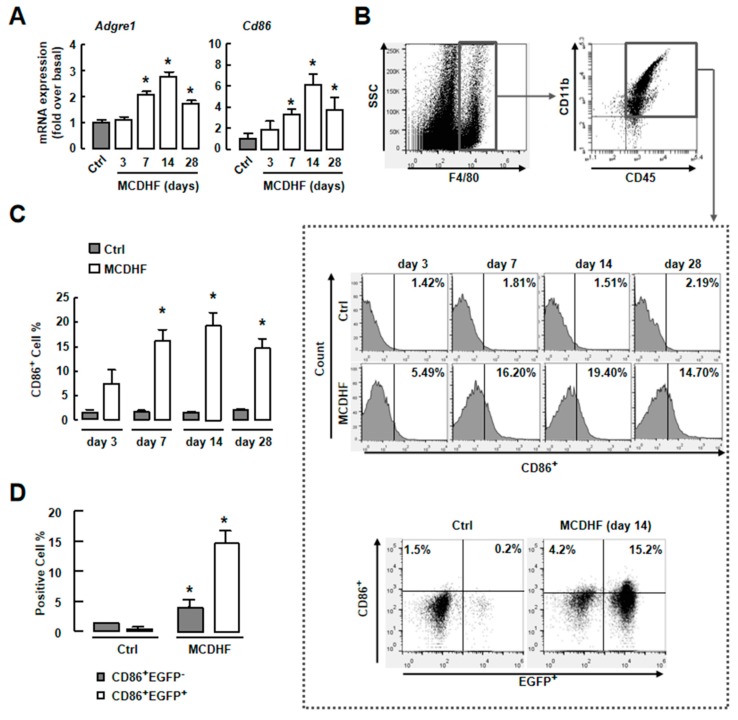
Increase of iMφ was associated with MCDHF-diet-induced liver injury in vivo. (**A**) The mRNA expressions of F4/80 (*Adgre1*) and *CD86* in the liver of MCDHF-diet-treated mice were determined by RT-qPCR. (**B**) Liver NPCs were isolated and gated by CD45, CD11b, and F4/80 in the liver of MCDHF-diet-fed mice (up). The total number of iMφ (CD86^+^) was measured in the liver of MCDHF-diet-treated mice from day 3 to day 28 (middle). The number of pro-inflammatory BMMs and pro-inflammatory KCs were measured in the liver of BM-transplanted mice after 14 days MCDHF diet feeding (low). (**C**) Quantification of total iMφ. (**D**) Quantification of pro-inflammatory BMMs and pro-inflammatory KCs. Data are presented as the mean ± SEM. * *p* < 0.05 versus control group (*n* = 6 for each group in each experiment).

**Figure 5:**
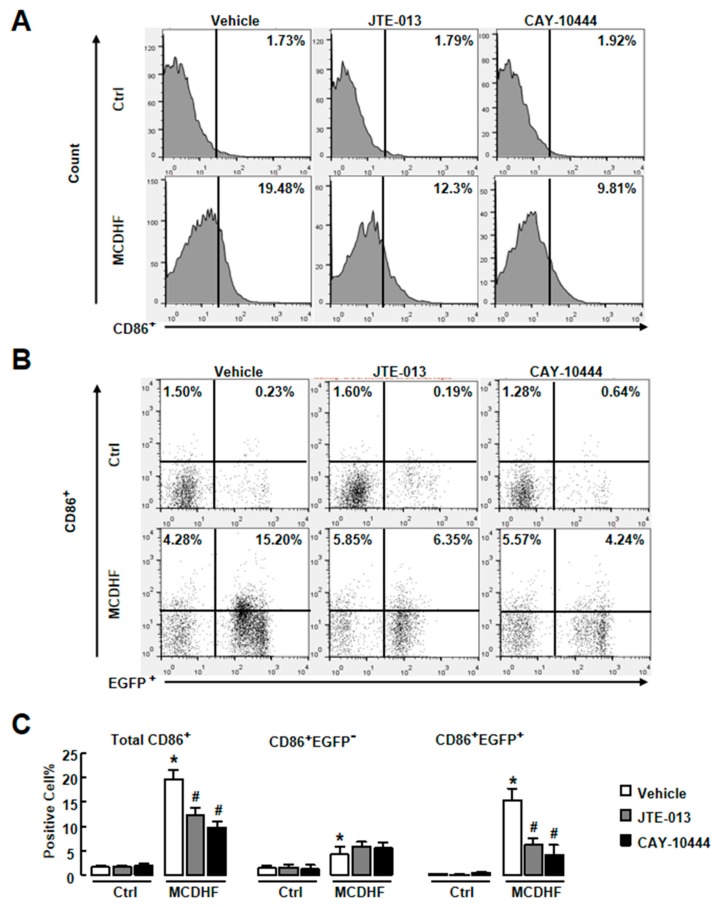
S1PR_2/3_ antagonist decrease the recruitment of iMφ in the liver of MCDHF diet-fed mice. Liver NPCs were isolated from vehicle, JTE-013-treated, and CAY-10444-treated mice after 14 days of control or MCDHF diet feeding. FACS was performed on these cells. (**A**) The percentage of total iMφ detected by FACS in the liver. (**B**) The percentage of pro-inflammatory BMMs or pro-inflammatory KCs in the liver. (**C**) Quantification of these cell components. Data are presented as the mean ± SEM. *p < 0.05 versus the group. # *p* < 0.05 versus the MCDHF diet group (*n* = 6 for each group in each experiment).

Studies have demonstrated that CD86^+^ macrophages are important producers of pro-inflammatory factors and play a key role in liver inflammation, injury, and fibrosis [26,27]. The results of RT-qPCR showed the increase of F4/80 *(Adgre1)* or *Cd86* expression in injured livers (Figure 4A). We next focused on iMφ activities in the fatty liver injury. First, we performed FACS to test the amount of iMφ in MCDHF-diet-fed mouse liver. Isolated hepatic NPCs were gated by CD45^+^CD11b^+^F4/80^+^ (macrophages) and then were examined for *CD86* expression (Figure 4B). The number of CD86^+^ cells increased at the beginning of day 7 to a maximal at day 14 (19.4%), suggesting that iMφ were associated with fatty liver injury (Figure 4B (middle), C). Second, we used an EGFP^+^ BM cell-transplanting mouse model to identify the origin of iMφ. The ratio of EGFP^+^ cells in the blood was calculated as control. It was unchanged in normal and injured mice (Supplementary Materials Figure S1B). After MCDHF diet feeding, the percentage of CD86^+^EGFP^+^ cells (pro-inflammatory BMMs) highly increased from 0.23% to 15.2%, whereas the proportion of CD86^+^EGFP^−^ cells (pro-inflammatory KCs) only increased from 1.5% to 4.2% (Figure 4B (low),D), indicating that iMφ in injured liver were mainly derived from BMMs. Third, we investigated whether S1PR_2/3_ affect the number of pro-inflammatory BMMs in injured liver. The MCDHF diet induced increase of iMφ in liver (19.48%) was prevented by JTE-013 (reduced to 12.3%) and CAY10444 (reduced to 9.81%) treatment, respectively (Figure 5A,C, left). Similarly, the number of pro-inflammatory BMMs decreased by JTE-013 or CAY10444 pretreatment in damaged livers of BM-transplanted mice, while the number of pro-inflammatory KCs did not changed (Figure 5B,C). These results prove that the decline of pro-inflammatory BMMs by specific S1PR_2/3_ antagonists contributes to the decrease of total iMφ.

**Figure 6:**
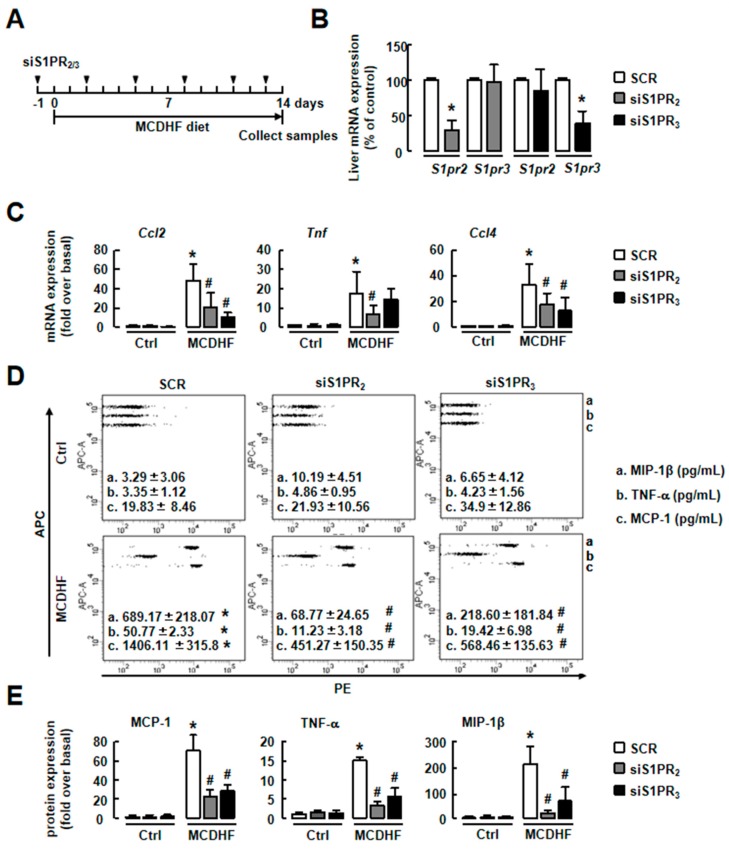
S1PR_2/3_ knockdown decreased inflammation in the liver of MCDHF-diet-fed mice. (**A**) Scheme of the mouse model. (**B**) The efficiency of S1PR_2_ or S1PR_3_ knockdown by their siRNAs in the liver. (**C**) The mRNA expressions of pro-inflammatory factors in the liver from SCR- and siS1PR_2/3_-treated mice after 14 days of control or MCDHF diet feeding. (**D**) The protein expressions of pro-inflammatory factors were examined by CBA. (**E**) Quantification of pro-inflammatory factor protein expressions. Data are presented as the mean ± SEM. * *p* < 0.05 versus the control group. # *p* < 0.05 versus the MCDHF diet group (*n* = 6 for each group in each experiment). SCR, scramble siRNA.

**Figure 7:**
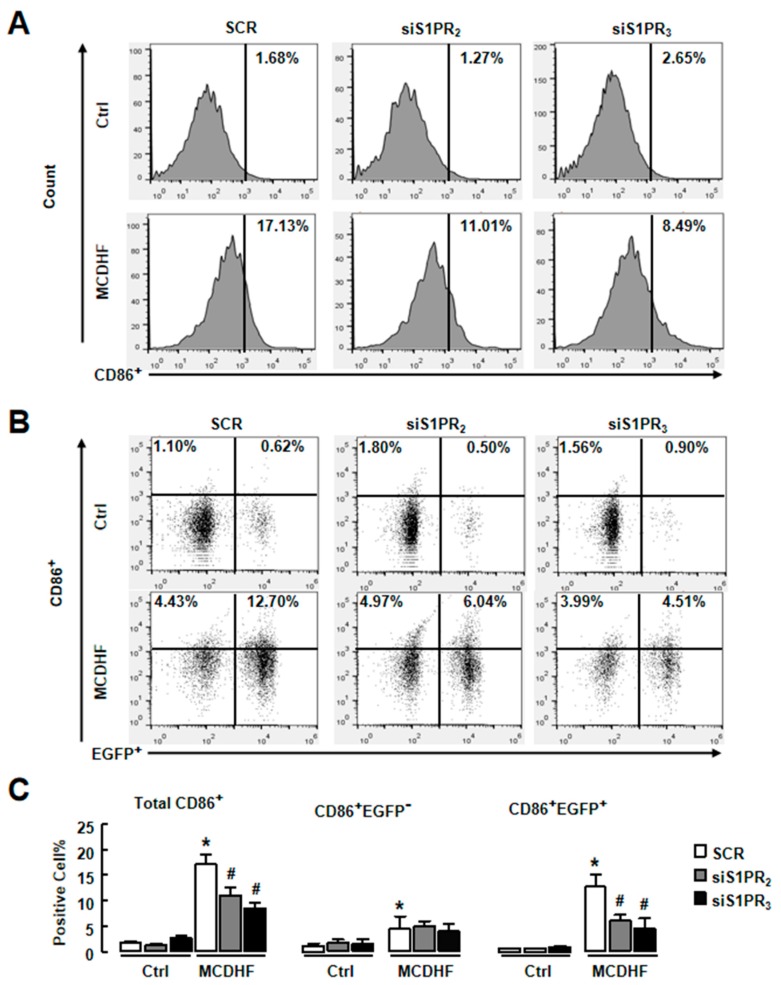
S1PR_2/3_ knockdown decreased iMφ recruitment in MCDHF-diet-fed mouse model. Liver NPCs were isolated from SCR- and siS1PR_2/3_-treated mice after 14 days of control or MCDHF diet feeding. FACS was performed on these cells. (**A**) The percentage of total iMφ detected by FACS in the liver. (**B**) The percentage of pro-inflammatory BMMs or pro-inflammatory KCs in the liver. (**C**) Quantification of these cell components. Data are presented as the mean ± SEM. * *p* < 0.05 versus the control group. # *p* < 0.05 versus the MCDHF diet group (*n* = 6 for each group in each experiment). SCR, scramble siRNA.

We also employed specific S1PR siRNAs to confirm these results. First, the efficiency of S1PR_2/3_ knockdown was tested. After injection of S1PR_2/3_ siRNAs, the hepatic S1PR_2/3_ levels were reduced as expected (Figure 6B). We also separated hepatocytes, NPCs or bone marrow cells and examined S1PR_2_ mRNA expression in MCDHF-diet-fed mice with or without S1PR_2_ knockdown. The results showed that S1PR_2_ expression decreased to ~15% in hepatocytes and ~20% in NPCs, while it was unchanged in bone marrow cells. Then, we detected the expressions of pro-inflammatory factors in S1PR_2/3_ siRNAs-pretreated injured livers. In accordance with S1PR_2/3_ antagonists, S1PR_2/3_ siRNAs decreased MCDHF-diet-induced pro-inflammatory factor productions (Figure 6C–E). Furthermore, S1PR_2/3_ siRNAs decreased the amount of iMφ, especially pro-inflammatory BMMs, in injured liver (Figure 7).

In a word, these results confirm the crucial role of S1PR_2/3_ in affecting the number of pro-inflammatory BMMs and hepatic inflammation in MCDHF-diet-fed mice. These results also suggest that S1PR_2/3_ blockade is an effective anti-inflammatory treatment in fatty liver injury.

### 2.4. Antagonism of S1PR_2/3_ Attenuates Hepatic Injury and Fibrosis, Not Fat Accumulation In Vivo

**Figure 8:**
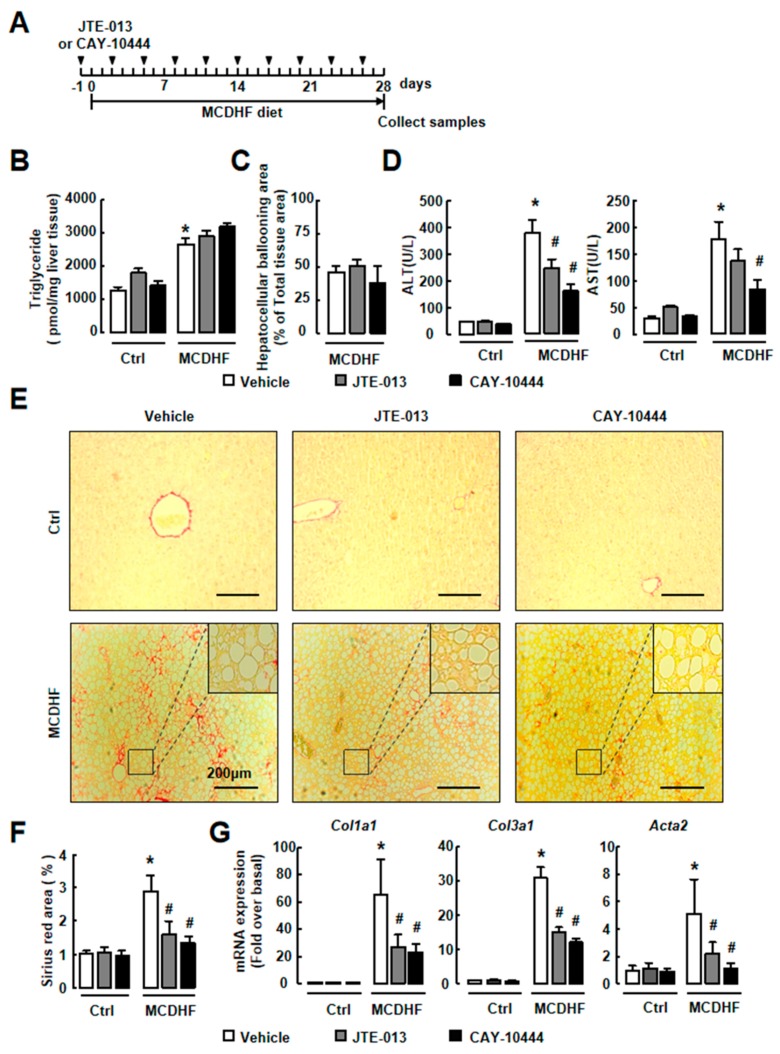
Antagonism of S1PR_2/3_ attenuated the hepatocellular injury and liver fibrosis in a MCDHF-diet-fed mouse model. Mice were fed with an MCDHF diet for 28 days with or without JTE-013 (10 mg/kg) or CAY-10444 (10 mg/kg) administration. (**A**) Scheme of the mouse model. (**B**) The level of liver triglyceride was measured in treated mice. (**C**) The area of hepatocellular ballooning was analyzed by calculating the percentage of the hepatocellular ballooning area in total liver tissue area. (**D**) The level of ALT and AST in the serum of treated mice. (**E**) The extent of fibrosis was determined by Sirius Red staining. Scale bars = 200 μm. (**F**) Quantification analysis of Sirius Red staining. (**G**) The mRNA expressions of *Col1a1*, *Col3a1*, and *α-SMA* (*Acta2*) in the liver were determined by RT-qPCR. Data are presented as the mean ± SEM. * *p* < 0.05 versus the control group, # *p* < 0.05 versus the MCDHF diet group without antagonists (*n* = 6 for each group in each experiment).

We next investigated whether S1PR_2/3_ blockade relieved MCDHF-diet-induced liver injury and fibrogenesis. Since the development of hepatic fibrosis requires a long-lasting MCDHF diet feeding, mice were fed with MCDHF diet for 28 days in these experiments. First, the role of S1PR_2/3_ on fat metabolism was studied. Liver TG was significantly increased in MCDHF-diet-treated livers and JTE-013 or CAY-10444 treatment did not affect it (Figure 8B). Furthermore, S1PR inhibitors did not reduce the area of hepatocellular ballooning (Figure 8C), indicating that the S1PR_2/3_ blockade did not affect fat accumulation in MCDHF-diet-induced fatty liver injury. Second, the serum levels of liver injury biomarkers, such as ALT and AST, were detected. We observed increases of serum ALT and AST in MCDHF-diet-treated mice and the increases were blocked by JTE-013 or CAY-10444 pretreatment (Figure 8D). Third, we determined the liver fibrogenesis by Sirius Red staining and found that the fibrotic areas were decreased significantly in the injured livers of mice treated with JTE-013 or CAY-10444 (Figure 8E–F). Further, the pictures of tissue biopsies also showed that steatosis was not affected by S1PR_2/3_ blockade in MCDHF-diet-treated livers (Figure 8E, inset). Fourth, we examined fibrogenesis marker expressions. There was also a reduction in the mRNA expression of *Col1a1*, *Col3a1* or α-SMA *(Acta2)* in the S1PR antagonist-treated mice (Figure 8G Supplementary Materials Figure S1F).

To further investigate the therapeutic effects of S1PR_2/3_ blockade, we fed mice with the MCDHF diet for 14 days, and then performed JTE-013 or CAY-10444 injection for another seven days (keeping the MCDHF diet). The results showed that S1PR_2/3_ inhibitors efficiently reduced MCDHF-diet-induced hepatic inflammation and fibrogenesis (Supplementary Materials Figure S2). Collectively, these results indicate that S1PR_2/3_ blockade exerts anti-inflammatory and anti-fibrotic effects in a mouse fatty liver injury model, suggesting that S1PR_2/3_ blockade could be an effective therapy for fatty liver injury.

## 3. Discussion

In the present study, we investigated whether S1PR_2/3_ blockade could be an effective treatment for fatty liver injury. The results provided evidence that the S1P/S1PR axis is an important modulator of the number of pro-inflammatory BMMs and pro-inflammatory factor production in the liver. The S1PR_2/3_ blockade works as an anti-inflammatory treatment and ameliorates liver inflammation, injury, and fibrosis in fatty liver injury.

The S1P/S1PR axis has been implicated in many key cellular processes, including cell proliferation, differentiation, migration, and survival [28]. Besides, the S1P/S1PR axis acts as a crucial inflammatory mediator and is activated in response to inflammation [16,29]. Our previous studies have reported that the S1P level is increased in liver tissues or serum in human or mouse liver fibrosis, while S1P level in bone marrow does not change. The increased S1P levels in injured liver and circulation form a concentration gradient which induces bone marrow cell recruitment towards injured liver [25,30,31]. The concentration gradient is set-up by the increase of the liver S1P level. Our recent studies have reported that S1PR_2/3_ are involved in pro-inflammatory activation of BMMs and increase pro-inflammatory factor production in vitro [9,24]. In this study, we proved that S1PR_2/3_ were potential therapeutic targets of fatty liver injury since the prophylactic or therapeutic S1PR_2/3_ blockade reversed liver inflammation, injury, and fibrosis in mice. However, the combined application of S1PR_2/3_ inhibitors did not show better effects than a single inhibitor, indicating S1PR_2/3_ did not work additively or synergistically.

In a recent study, researchers found that FTY720 (a broad-spectrum S1PR modulator against S1PR_1/3/4/5_) ameliorates non-alcoholic steatohepatitis in mice by reducing hepatic macrophage accumulation [32]. In vitro, specific knockdown of S1PR_1_ in saturated fat-treated HepG2 cells downregulates the expression of inflammatory factors [33]. These reports indicate that S1PR_1_ is also a regulator of hepatic inflammation and fatty liver injury. However, the roles of S1PR_1_ and S1PR_2/3_ are different. S1PR_1_ works mainly on hepatocytes and influences macrophage recruitment indirectly, while S1PR_2/3_ affect macrophage functions directly. Besides BMMs, S1PR_2/3_ also regulate other immune cell functions. For example, S1PR_2_ affects mast cell triggering during viral infection. S1PR_3_ also affects dendritic cell maturation and neutrophil or eosinophil recruitment [16]. During liver injury, the S1P/S1PR axis also participates in the functions of other hepatic NPCs. For example, S1PR_1/3_ mediate S1P-induced human hepatic stellate cells migration, while S1PR_2_ plays an anti-migratory manner in this process [31]. S1PR_3_ is involved in bone marrow derived mesenchymal stem cells migration during liver fibrosis [25,30]. S1PR_1/3_ also play a role in ECM production in liver fibrosis [15]. Our present data proved that the S1PR_2_ level was much higher in macrophages than other NPCs in injured livers, suggesting macrophages might be more sensitive to the S1PRs blockade. At the same time, the upregulations of S1P and the S1PR_2/3_ levels appeared at early stage of fatty liver injury (S1P at 7 days, S1PR_2_ at 14 days, S1PR_3_ at 3 days). Macrophage amount and pro-inflammatory factor production were also increased at this stage (peak at 14 days). Herein, we concluded that S1PR_2/3_ blockade mainly affected macrophage functions in mice within 14 days of feeding them the MCDHF diet. However, it is still worth it to further study how the S1PR blockade performs its powerful therapeutic effects in liver injury.

Fatty liver injury is the result of lipid accumulation in hepatocytes [2]. Several lines of evidence have indicated that hepatocellular lipid accumulation promotes the hepatocyte susceptibility for diverse pro-apoptotic stimuli and elicits hepatocyte apoptosis or necrosis [34,35]. This phenomenon is referred to as hepatic lipotoxicity and is believed to play a crucial role in the pathogenesis of liver inflammation [36]. Hepatic inflammation is considered the major driver of liver tissue injury leading to fibrogenesis [4,5]. In liver, inflammatory response is attributed to the innate immune system, which is characterized by recruitment of various inflammatory cells to the liver, such as macrophages [37]. Studies have shown that macrophages are crucial players in metabolic homeostasis [38,39]. They respond to metabolic signaling and release inflammatory cytokines to initiate and maintain liver inflammation [40]. Some studies have demonstrated that activated macrophages play a pro-fibrotic role in liver diseases [10]. Therefore, anti-inflammatory treatment, especially controlling the number of liver macrophages or functions during inflammatory response, is considered as an effective therapy of liver diseases. In a recent research, therapeutic inhibition of inflammatory monocytes recruitment by CCR2/CCR5 antagonist reduces steatohepatitis and liver fibrosis in mice [41]. Our previously studies also demonstrated that inhibition of iMφ activation relieves liver inflammation or injury [9,42]. Here, we provided new evidence for the role of iMφ in fatty liver injury and proved the importance of anti-inflammatory therapy for liver diseases. We found that anti-inflammatory treatment (S1PR_2/3_ blockade) reversed fatty liver injury, although the abnormal fat accumulation still existed.

Macrophages are major cellular component of the innate immune and liver injury [43]. Hepatic macrophages are composed of KCs and BMMs. Studies have shown that KCs are critical contributors in alcoholic hepatic steatosis-induced liver inflammation and injury [44,45]. At the same time, accumulating data, including our previous data, suggest that BMMs are also implicated in various liver inflammation responses [7,9,42,46,47]. Here, we revealed that a significant proportion of iMφ (CD86^+^) was of BM origin (BMMs, EGFP^+^) following MCDHF-diet-induced liver injury, and blockade of S1PR_2/3_ decreased the amount of pro-inflammatory BMMs in injured liver.

In summary, we identified the important role of S1PR_2/3_ in fatty liver injury and demonstrated that S1PR_2/3_ blockade inhibits liver injury in vivo, strongly suggesting S1PR_2/3_ might serve as therapeutic targets for fatty liver injury. Our data open up a comprehensive understanding of fatty liver injury process and provide potential targets for rational development of therapeutic drugs.

## 4. Materials and Methods

### 4.1. Materials

The RPMI 1640 Medium was obtained from Invitrogen (Grand Island, NY, USA). Fetal bovine serum was purchased from Biochrom (Berlin, Germany). The PCR reagents were bought from Applied Biosystems (Foster City, CA, USA). Histopaque-1077 and other common reagents were purchased from Sigma (St. Louis, MO, USA). The JTE-013 and CAY-10444 were obtained from Cayman Chemical (Ann Arbor, MI, USA).

### 4.2. Animal Model

Male ICR mice, 31.0 ± 1.0 g, at six weeks of age were fed either control diet (Ctrl) or MCDHF diet (Research Diet, New Brunswick, NJ, USA) containing 46 kcal% fat, 18 kcal% protein, and 36 kcal% carbohydrate. At day 3, 7, 14, and 28 after MCDHF diet feeding, mice were anesthetized to collect samples. The i.p. injection of JTE-013 (10 mg/kg body weight), CAY-10444 (10 mg/kg body weight) or vehicle was performed 24 h before MCDHF diet feeding, then twice injected with the same dose per week. For evaluating the therapeutic effects of JTE-013 or CAY-10444, mice were fed with an MCDHF diet for 14 days and/then were i.p. injected with these inhibitors or vehicle for another 7 days (*n* = 6). The liver tissues or serum samples were collected for further analysis. All animal work conformed to the Ethics Committee of Capital Medical University and in accordance with the approved guidelines (approval number: AEEI-2014-131).

### 4.3. BM Transplantation

The ICR mice received lethal irradiation (8 Grays in a divided dose 4 h apart), and immediately received transplantation by a tail vein injection of 1.5 × 10^7^ whole BM cells which obtained from EGFP transgenic mice. Four weeks later, BM was rebuilt, and the chimera mice were with EGFP-labeled BM cells. Then, the chimera mice were used in animal experiment as described above.

### 4.4. RNA Interference (RNAi) In Vivo

Mouse chemically modified and stable small interfering RNA (siRNA) of S1PR_2_ (UAA CUC CCG UGC AGU GGU UUU) and S1PR_3_ (GGA GGG CAG UAU GUU CGU AUU) were purchased from Thermo Scientific (Lafayette, CO, USA), and were delivered in vivo using a “hydrodynamic transfection method”, by which 50 μg siRNA dissolved in 1 mL PBS was rapidly injected into the tail vein. Control mice were injected with an equal volume of scramble (SCR) siRNA dissolved in PBS. These siRNAs were injected one day before MCDHF-diet-induced liver injury, and twice every 7 days after MCDHF diet feeding for 14 days.

### 4.5. Fluorescence-Activated Cell Sorting (FACS)

The NPCs of mouse liver were isolated from chimera mice, then analyzed by flow cytometry as described previously [42]. To make gating strategies for analysis of macrophages, liver NPCs were stained with APC-conjugated anti-F4/80 Ab (BD Bioscience, Franklin Lakes, NJ, USA), eFluor 450-conjugated anti-CD45 (eBioscience, CA, USA), FITC-conjugated anti-CD11b (BD Bioscience, USA) or PE-conjugated anti-CD86 Ab (BD Bioscience, USA) for 15 min at 4 °C in the dark. The analysis was performed using FACS Calibur H (BD Bioscience).

### 4.6. S1P Quantitation

The concentration of hepatic S1P was measured by the S1P ELISA kit (Echelon, USA) according to the manufacturer’s instructions. A standard curve was created, and the results were normalized to the protein content of the sample (pmol S1P/mg protein).

### 4.7. RT-qPCR

The RT-qPCR was performed as described previously [42]. All primers were synthesized by AuGCT Biotechnology (Beijing, China). Primers used for RT-qPCR were as follows: 18S rRNA: sense, 5′-GTA ACC CGT TGA ACC CCA TT-3′; antisense, 5′-CCA TCC AAT CGG TAG TAG CG-3′. F4/80 (*Adgre1*): sense, 5′-AGC ACA TCC AGC CAA AGC A-3′; antisense, 5′-CCA TCT CCC ATC CTC CAC AT-3′; *Cd86*: sense, 5′-TCC AAG TTT TTG GGC AAT GTC-3′; antisense, 5′-CCT ATG AGT GTG CAC TGA GTT AAA CA-3′. The MIP-1β (*Ccl4*): sense, 5′-CCA GCT CTG TGC AAA CCT AAC C-3′; antisense, 5′-GCC ACG AGC AAG AGG AGA GA-3′. TNF-α (*Tnf*): sense,5′-GGC AGG TTC TGT CCC TTT CA-3′; antisense, 5′-CTG TGC TCA TGG TGT CTT TTC TG-3′. MCP-1 (*Ccl2*): sense, 5′-TCT GGG CCT GCT GTT CAC A-3′; antisense, 5′-GGA TCA TCT TGC TGG TGA ATG A-3′. α-Smooth muscle actin (α-SMA, *Acta2*): sense: 5′-ATG CTC CCA GGG CTG TTT T-3′; antisense: 5′-TTC CAA CCA TTA CTC CCT GAT GT-3′. Procollagen α1(I) (Colα1(I), *Col1a1*): sense: 5′-AGG GCG AGT GCT GTG CTT T-3′; antisense: 5′-CCC TCG ACT CCT ACA TCT TCT GA-3′. procollagen α1(III) (Colα1(III), *Col3a1*): sense: 5′-TGA AAC CCC AGC AAA ACA AAA-3′; and antisense, 5′-TCA CTT GCA CTG GTT GAT AAG ATT AA-3′. *S1pr1*: sense: 5′-ACT TTG CGA GTG AGC TG-3′; antisense: 5′-AGT GAG CCT TCA GTT ACA GC-3′. *S1pr2*: sense: 5′-TTC TGG AGG GTA ACA CAG TGG T-3′; antisense, 5′-ACA CCC TTT GTA TCA AGT GGC A-3′. *S1pr3*: sense: 5′-TGG TGT GCG GCT GTC TAG TCA A-3′; antisense, 5′-CAC AGC AAG CAG ACC TCC AGA-3′. *S1pr4*: sense: 5′-TGC GGG TGG CTG AGA GTG-3′; antisense, 5′-TAG GAT CAG GGC GAA GAC C-3′. *S1pr5*: sense: 5′-CTT AGG ACG CCT GGA AAC C-3′; antisense, 5′-CCC GCA CCT GAC AGT AAA TC-3′. *Sphk1*: sense: 5′- TGT CAC CCA TGA ACC TGC TGT CCC TGC ACA-3′; antisense: 5′- AGA AGG CAC TGG CTC CAG AGG AAC AAG-3′. *Sphk2*: sense: 5′- ACA GAA CCA TGC CCG TGA G-3′; antisense: 5′-AGG TCA ACA CCG ACA ACC TG-3′. Probes (Applied Biosystems, Foster City, CA, USA) used for RT-qPCR were as follows: *Sgpp1*: MA00473016 and *Sgpl1*: MA00486079.

### 4.8. Histology Analysis

Liver tissues were fixed in 4% buffered formaldehyde. Liver tissue sections (5 μm) were stained with H&E staining for assessment of inflammation and injury and Sirius Red staining for the extent of collagen deposition. To quantify the degrees of liver injury, histology activity index (HAI) was employed [48]. The number of hepatic inflammatory foci or area of fibrosis was measured in three sections. The fibrotic areas were assessed as previously described [42]. Morphometric analysis of H&E and Sirius Red staining was done using ImageJ 1.4 (NIH).

### 4.9. Measurement of Cytokine and Chemokine by CBA

Liver tissues (~40 mg) homogenate was lysed in 40 μL lysis buffer. The MIP-1β, TNF-α, and MCP-1/CCL2 in homogenates were detected using CBA mouse MIP-1β Flex Set, mouse TNF-α Flex Set, and mouse MCP-1/CCL2 Flex Set (BD Biosciences). Samples or standards were mixed with a combination of different fluorescence intensity beads and detection cytokine antibodies conjugated to PE, and then analyzed by flow cytometry. The pro-inflammatory factor concentrations were calculated through comparison with the standard curve of each pro-inflammatory factor.

### 4.10. Biochemical Assays

Serum was separated and stored at −80 °C until use. Serum alanine aminotransferase (ALT) and aspartate aminotransferase (AST) were measured using commercial assay kits (Stanbio, Boerne, TX, USA).

### 4.11. Triglyceride Assay

Liver triglyceride (TG) was assayed using a TG assay kit (Applygen Technologies Inc., Beijing, China) according to the manufacturer’s recommended protocol.

### 4.12. Statistical Analysis

The results are expressed as mean ± SEM from at least three independent experiments. Statistical significance was assessed by Student’s *t*-test or ANOVA for analysis of variance when appropriate. Correlation coefficients were calculated by a Pearson test. *p* < 0.05 was considered to be significant.

## Supplementary Materials

Supplementary materials can be found at https://www.mdpi.com/1422-0067/20/19/4695/s1.
