# Supplementary material for: Sphingosine 1-Phosphate Receptor Blockade Affects Pro-Inflammatory Bone Marrow-Derived Macrophages and Relieves Mouse Fatty Liver Injury
Source: Int J Mol Sci. 2019 Sep 22;20(19):4695. doi: 10.3390/ijms20194695 (PMC6801987; doi:10.3390/ijms20194695)
Supplement: Supplementary file 1 [file ijms-20-04695-s001.zip › Supplementary figures/Supplementary figure 1.pdf]

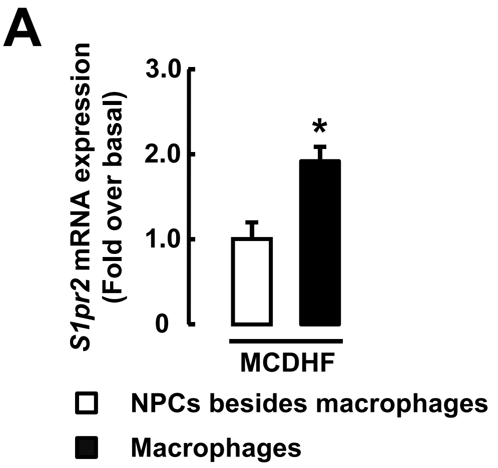

B

	EGFP+ cell count	Total cell count	Ratio of EGFP+ cell
Ctrl	397	574	69.13%
MCDHF diet	475	657	72.39%

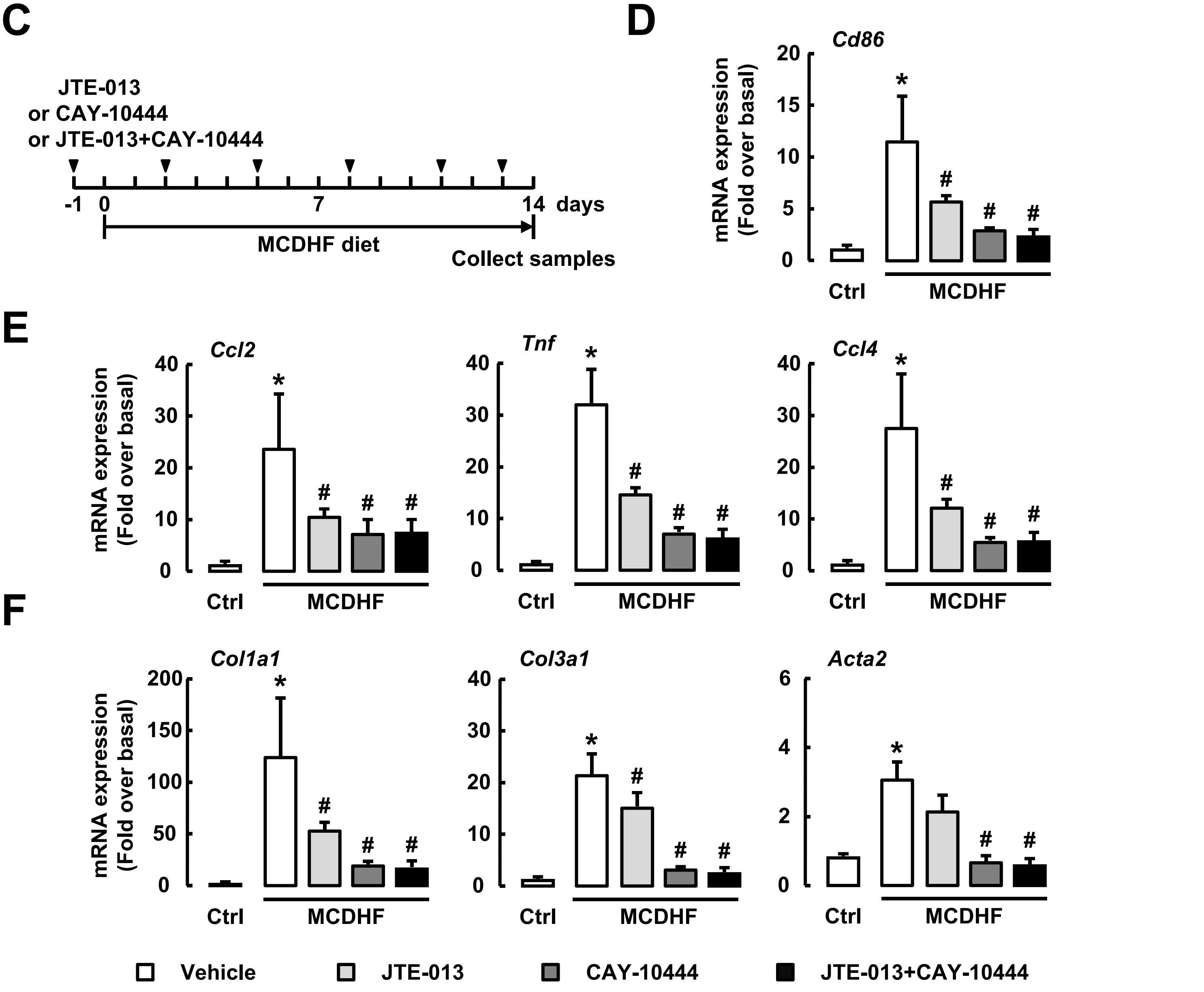

Supplemental Figure 1. S1PR2/3 blockade decrease hepatic inflammation and fibrogenesis in the MCDHF diet-fed mice.

(A) MCDHF diet-fed mice were subjected to liver perfusion with 0.4% collagenase buffer. NPCs were separated using low-speed centrifugation and 40% percoll density gradient centrifugation. F4/80 was used to sorted macrophages using FACS. Then S1pr2 mRNA expression in macrophages or remaining NPCs were examined. (B) The ratio of EGFP+ cells in the blood. (C) Scheme of the mouse model. (D-F) The mRNA expressions of Cd86 (D), pro-inflammatory factors (E), and fibrotic genes (F) in the liver from vehicle, JTE-013-, CAY-10444-, and JTE-013+CAY-10444-treated mice after 14 days of Ctrl or MCDHF diet feeding. Data are presented as the means ± SEM. **P*<0.05 vs. Ctrl group. # *P*<0.05 vs. MCDHF diet group (n=6 for each group in each experiment).
