# Supplementary material for: Sphingosine 1-Phosphate Receptor Blockade Affects Pro-Inflammatory Bone Marrow-Derived Macrophages and Relieves Mouse Fatty Liver Injury
Source: Int J Mol Sci. 2019 Sep 22;20(19):4695. doi: 10.3390/ijms20194695 (PMC6801987; doi:10.3390/ijms20194695)
Supplement: Supplementary file 1 [file ijms-20-04695-s001.zip › Supplementary figures/Supplementary figure 2.pdf]

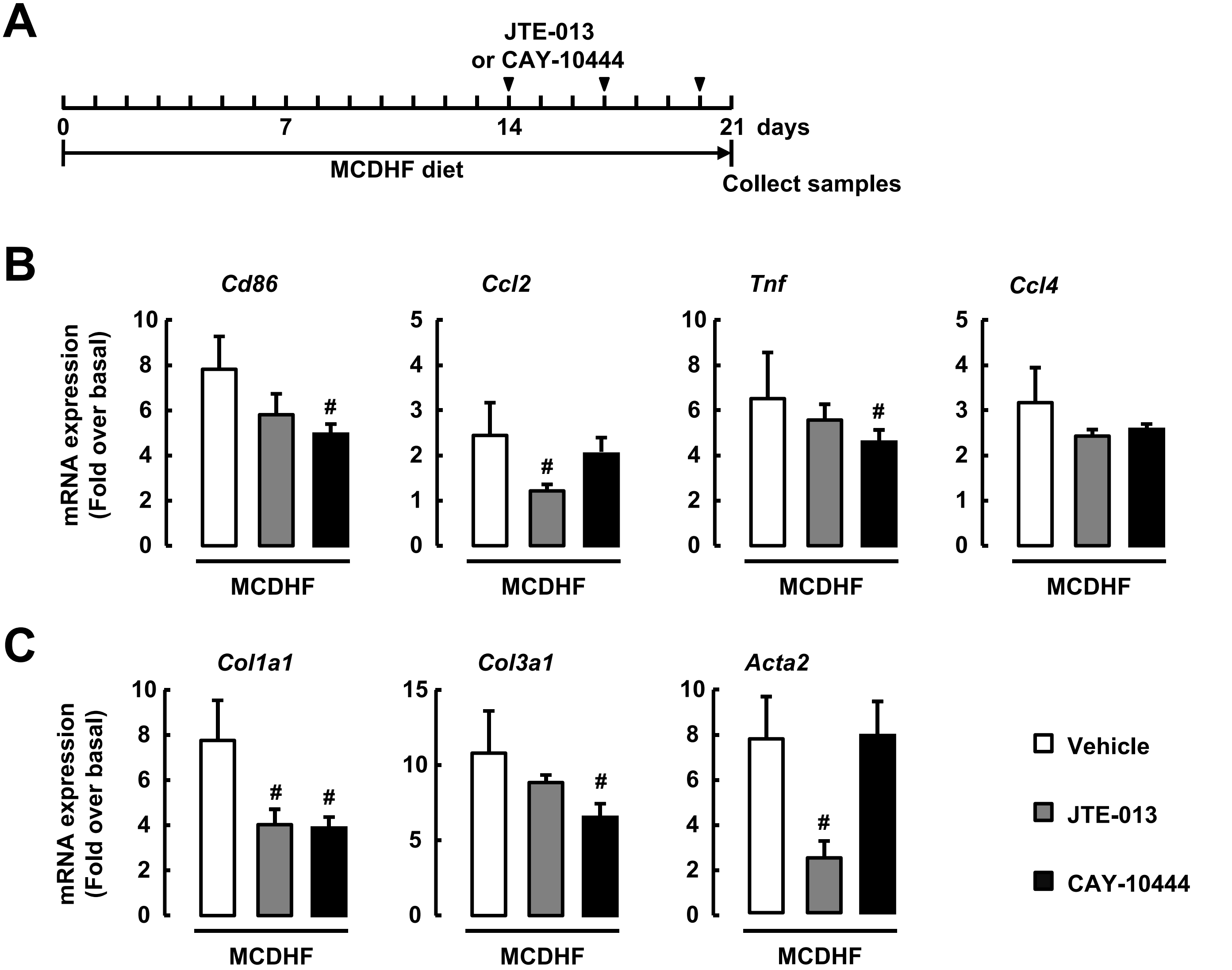

Supplemental Figure 2. Therapeutic injection of S1PR2/3 inhibitors decreases hepatic inflammation and fibrogenesis in the MCDHF diet-fed mice.

(A) Scheme of the mouse model. (B) The mRNA expressions of pro-inflammatory factors in the liver from vehicle, JTE-013-, CAY-10444-treated mice after 14 days of Ctrl or MCDHF diet feeding. (C) The mRNA expressions of fibrotic genes in the liver from vehicle, JTE-013-, CAY-10444-treated mice after 14 days of Ctrl or MCDHF diet feeding. Data are presented as the means ± SEM. **P*<0.05 vs. Ctrl group. # *P*<0.05 vs. MCDHF diet group (n=6 for each group in each experiment).
